# The effect of a sand surface on physical performance responses of junior male handball players to plyometric training

**DOI:** 10.1186/s13102-020-00176-x

**Published:** 2020-04-25

**Authors:** Mehrez Hammami, Nicola Luigi Bragazzi, Souhail Hermassi, Nawel Gaamouri, Ridha Aouadi, Roy J. Shephard, Mohamed Souhaiel Chelly

**Affiliations:** 1 Research Unit (UR17JS01) « Sport Performance, Health & Society», Higher Institute of Sport and Physical Education of Ksar Saîd, University of “La Manouba”, Tunis, Tunisia; 2 Higher Institute of Sport and Physical Education of Ksar Said, University of “La Manouba”, Tunis, Tunisia; 3 Laboratory for Industrial and Applied Mathematics (LIAM), Department of Mathematics and Statistics, York University, Toronto, ON Canada; 4 Postgraduate School of Public Health, Department of Health Sciences (DISSAL), University of Genoa, Genoa, Italy; 5 Sport Science Program, College of Arts and Sciences, Qatar University, Doha, Qatar; 6 Faculty of Kinesiology and Physical Education, University of Toronto, Toronto, ON Canada

**Keywords:** Sand, Dynamic balance, Agility, Stretch shortening cycle, Sprint performance

## Abstract

**Background:**

The effects of 7 weeks plyometric training on a stable surface and on sand were compared in junior male handball players.

**Methods:**

A team of experienced players was divided randomly between three groups, undertaking a standard in-season regimen (C, *n* = 10), or supplementing this regimen by plyometric training on sand (PS, *n* = 11) or a stable surface (P, n = 10) for 7 weeks. Assessments included 20 m sprint times, change of direction tests (Modified change-of-direction T-test and Modified Illinois test), a repeated sprint T-test, jumping ability (squat, countermovement and five jump tests), and static and dynamic balance.

**Results:**

After the intervention, PS showed significant increases of sprint speed relative to P and C. Change of direction scores were also improved for PS relative to P and C. Both PS and P increased vertical jump performance (squat jump, *p* = 0.005; ES = 0.170; counter-movement jump *p* < 0.001; ES = 0.247). Repeated sprint T-test scores improved in PS and P relative to C, with best times of PS > P (*p* < 0.05). Both plyometric groups improved their dynamic balance (p < 0.05), with three parameters of PS and only one of P being significantly greater than C. Static balance was also enhanced in both experimental groups (PS > P).

**Conclusions:**

We conclude that for reasons that remain to be clarified, several performance measures in adolescent male handball players were increased more by 7 weeks of PS than by P.

## Background

Time and motion analyses have demonstrated that in a typical game of handball there are 190 movement variations, 279 changes-of-direction, and 16 jumps with a total of 485 high-intensity actions [1, 2], in response to visual and/or auditory cues [1, 2]. Players must change direction with a minimum loss of speed, balance, and/or motor control, and make short, maximal efforts with only brief recovery periods. Plyometric activity is a natural part of this sport [3, 4]. Hammami et al. [5] reported increases in sprint, change-of-direction and jump performance after 8 weeks of plyometric training combined with change of direction exercises in U15 male handball players, and Dello Iacono et al. [1] found that plyometric training enhanced both horizontal and vertical jumps in elite male handball players (8.5 and 4% respectively). However, it remains of interest whether such gains could be enhanced by the use of an unstable training surface.

Some research has compared the effectiveness of training on stable and unstable surfaces. Negra et al. [6] and Granacher et al. [7] found comparable gains on measures of muscle power (e.g., countermovement and standing jumps, sprint speeds, dynamic balance, and agility tests) on stable vs. unstable surfaces in pre-pubertal male soccer players. Arazi et al. [8] also observed similar improvements in vertical jumps [4 (ES = 0.63) vs. 5.4 (ES = 0.85) cm], standing long jumps [8.3 (ES = 0.3) vs. 12.7 (ES = 0.57) cm], and 1RM leg press [23.5 (ES = 0.56) vs. 15.3 (ES = 0.49) kg] for sand and land-drop training. Likewise, these authors noted comparable decreases in 20-m [0.3 (ES = 0.72) vs. 0.4 (ES = 1.98) s], 40-m sprint times [0.2 (ES = 0.4) vs. 0.5 (ES = 0.71) s], and T-test scores [0.5 (ES = 0.62) vs. 0.9 (ES = 0.57) s] for sand and land-drop jump groups [8]. Ramirez-Campillo et al. [9] compared responses to plyometric training (7 weeks, 2 sessions per week) when performed on a wooden gymnasium floor or an unstable 3-cm thick athletic mat, looking at measures of strength; however, their findings were inconsistent with respect to the effects of training surface [9].

Thus, several reports have found little advantage from the use of unstable surfaces. However, as yet few studies have compared muscular performance responses on sand and firm surfaces. Arazi et al. [8] observed that training on sand enhanced agility and strength relative to standard plyometrics, and Impellizzeri et al., [10] noted gains of sprinting, jumping and sprinting ability with less muscle soreness when their participants trained on sand rather than on grass. The present study compared gains in the muscular performance of male handball players after 7 weeks of plyometric training on either sand (PS) or a normal firm (Gymnasium floor) surface (P). We hypothesized that gains in performance would be greater for PS than for P.

## Methods

### Participants

Experienced players were divided between three groups: standard plyometrics performed on a gymnasium floor (P, *n* = 10), plyometrics on a dry sandy surface (PS, *n* = 11), and controls (C, n = 10) (Table 1). All participants completed two familiarizations trials of all except anthropometric measurements in the 2 weeks before definitive data collection. Baseline testing was undertaken in the last 2 weeks of July, and tests were repeated after the 7-week intervention. The test protocol comprised a 20 m sprint test, change-of-direction tests (Modified agility T-test, Modified Illinois test, and repeated change of direction test (RSTT)), jumping (squat, counter-movement and five jump tests) and Stork and Y-balance tests. Measurements were made at a consistent time of day and under the same experimental conditions, at least 3 days after the most recent competition and (for the final tests) 5–9 days after completion of the intervention. A normal intake of food and fluids was maintained except that participants drank no caffeine-containing beverages for 4 h and ate no food for two hours prior to testing. Verbal encouragement ensured maximal effort throughout.

**Table 1:** Physical characteristics of experimental and control groups (mean ± SD)

	Age (years)	Body mass (kg)	Height (m)	% Body fat
PS (n = 11)	16.2 ± 0.6	70.8 ± 7.3	1.80 ± 0.03	19.6 ± 4.2
P (n = 10)	16.4 ± 0.5	69.7 ± 6.9	1.78 ± 0.07	20.2 ± 8.9
C (n = 10)	16.5 ± 0.4	70.5 ± 5.7	1.79 ± 0.06	18.4 ± 3.6

PS = plyometrics on sand; P = standard plyometrics; C = control group; n = number

### Experimental design

Written informed consent was obtained from all participants and their parents or guardians before participating in a study approved by the Local Ethics Committee Research Unit (UR17JS01) “Sport Performance, Health & Society”, University of “La Manouba” in conformity with principles identified in the Declaration of Helsinki. The participants (31 junior male handball players, elite-level championship) were told that they could withdraw from the trial without penalty at any time. All were examined by the team physician, with a particular focus on orthopedic and other conditions that might preclude resistance training and all were found to be in good health. The three groups were well matched in terms of their initial physical characteristics.

### Testing procedures

All participants engaged in training sessions, supervised by the team coaches from the beginning of physical preparation (July) until conclusion of the trial (September). All engaged in handball training 6–7 times per week and played one friendly game per week. Standard training sessions lasted 90–100 min; usually, these emphasized the development of skills at various intensities, offensive and defensive strategies, and 25 to 30 min of continuous play, with only brief interruptions by the coach.

### Details of plyometric training

Both plyometric groups undertook an identical program every Tuesday, Thursday and Saturday for 7 consecutive weeks, P performing plyometrics on gymnasium floor and PS on dry sand; they replaced 25 min of their standard regimen (the technical-tactical skill development) by the intervention on those days. The plyometric training program for PS and P consisted of four principal workshops (Table 2). Each workshop began with plyometric exercises (hopping, lateral hurdle jumps, horizontal jumps, hurdle jumps) and finished with a 10 m linear sprint. Sessions began with a 10-min warm-up and lasted 35 min (Table 2), always supervised by the same coach; there were from 54 to 108 ground contacts per session. Verbal encouragement ensured a high level of motivation throughout.

**Table 2:** Components of plyometric training for the two experimental groups

Week	Workshop 1	Workshop 2	Workshop 3	Total contacts
1	6 × 3	6 × 3	6 × 3	54
2	6 × 4	6 × 4	6 × 4	72
3	6 × 4	6 × 4	6 × 4	72
4	6 × 5	6 × 5	6 × 5	90
5	6 × 5	6 × 5	6 × 5	90
6	6 × 6	6 × 6	6 × 6	108
7	6 × 6	6 × 6	6 × 6	108

Workshop 1 = 6 lateral 0.3 m hurdle jumps (3 to left and 3 to right), then sprinting 10 m; Workshop 2 = 6 horizontal jumps (3 to left and 3 to right), then sprinting 10 m; workshop 3 = 6 × 0.4 m hurdle jumps, then sprinting 10 m

### Anthropometry

Measurements included height and sitting height (accuracy of 0.1 cm; Holtain stadiometer, Crosswell, Crymych, Pembs, UK) and body mass (0.1 kg; Tanita BF683W scales, Munich,Germany). The overall percentage of body fat was estimated from the biceps, triceps, subscapular, and suprailiac skinfolds, using the equation of Durnin and Womersley for adolescent males aged 16.0–19.9 years [11].

### Sprint performance

Sprinting began with a standardized 20-min warm-up. Participants then ran 20 m from a standing position, with times recorded by paired photo cells (Microgate, Bolzano, Italy) set at 5, 10, and 20 m. Three trials were separated by 6–8 min of recovery, with the best results being recorded. Test-retest reliability and 95% confidence intervals over 5, 10 and 20 m distances were 0.857, 0.869, 0.879 and 0.847–0.891, 0.836–0.887, 0.758–0.902 respectively.

### Vertical jumping

After a 15-min warm-up, flight times (precision 0.001 s) and thus jump heights were assessed using an infrared photocell mat and digital computer (Optojump System, Microgate SARL, Bolzano, Italy). Details of squat and counter-movement jump technique have been described previously [5]. Test-retest reliability and 95% confidence interval for the two measures were 0.921, 0.923 and 0.781–0.941, 0.807–0.958 respectively.

### Five-jump test

Participants covered as much distance as possible with 5 forward jumps [5]. Test-retest reliability and 95% confidence intervals for this measure were 0.827 and 0.748–0.891 respectively.

### Modified change-of-direction T-test

The modified change-of-direction T-test determined speed with super-imposed directional changes (forward sprinting, left and right shuffling, and backward running) [12]. Performance times were recorded by paired photocells (Microgate, Bolzano, Italy); test-retest reliability and 95% confidence interval were 0.924 and 0.815–0.954 respectively.

### Modified Illinois test

Details of this agility test have previously been published [13]. Performance times were recorded by paired single beam photocells (Microgate, Bolzano, Italy); the test-retest reliability and 95% confidence interval for this measure were 0.906 and 0.783–0.921 respectively.

### Repeated sprint T-test

This test offers a reliable and valid measurement of the ability to change directions rapidly, simulating a game with short, intense efforts, recovery periods and multi-directional displacements [14]. Measurements included best time, mean time, total time and a fatigue index calculated as = ((Total time / (Best time × 7)) × 100) – 100 [15].

### Stork test of static balance

The Stork Test was performed in the standard manner [6], with participants standing on their dominant leg and resting their opposite foot against the inside of the supporting knee. Test -retest reliability scores for measurements on the right leg and left legs were 0.784 and 0.773, with respective 95% confidence interval of 0.409–0.847 and 0.617–0.819.

### Dynamic balance

Dynamic balance was assessed on the dominant leg, using the Y-balance test [6]. Three trials were conducted in each direction, with two-minute rest intervals. Test-retest reliabilities for the 3 reach directions ranged from 0.869 to 0.911, with respective 95% confidence intervals of 0.783–0.916, 0.814–0.898, 0.784–0.921 for the left, back and right side respectively (right support leg); and 0.845–0.956, 0.874–0.926, 0.805–0.911 for the left, back and right side respectively (left support leg).

### Statistical analyses

Statistical analyses were carried out using the Statistical Package for the Social Sciences (SPSS) version 23 program for Windows (SPSS, Inc., Chicago, IL, USA). Training-related effects were assessed by 2-way analyses of variance (group x time). If a significant F value was observed, Tukey’s *posthoc* procedure was applied to locate pair-wise differences. We accepted *p* ≤ 0.05 as our criterion of statistical significance, whether a positive or a negative difference was seen. Effect sizes were determined by converting partial eta-squared to Cohen’s d [16]; values were classified as small (0.00 ≤ d ≤ 0.49), medium (0.50 ≤ d ≤ 0.79), and large (d ≥ 0.80). Percentage changes were calculated as ([post-training value - pre-training value]/pre-training value) × 100. The reliabilities of measurements were assessed using intra-class correlation coefficients (ICC) [17], all reached an acceptable level of reliability (r > 0.80).

## Results

**Fig. 1:**
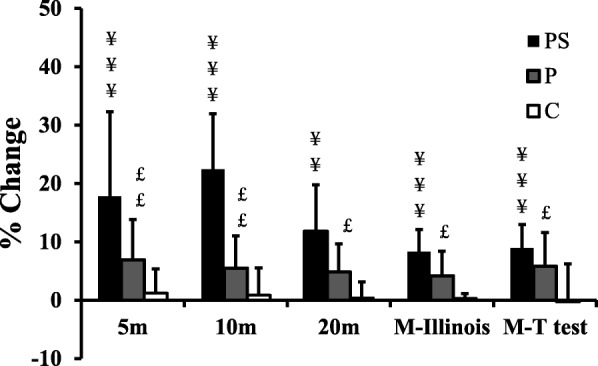
Training associated changes in sprint performance and ability to change direction following plyometric training on sand (PS), plyometric training on a stable surface (P) and controls following the standard regimen (C). M-Illinois: Modified Illinois test; M-T test: Modified change-of-direction T-test; ¥ = denotes a significant difference between PS and C; £ = denotes a significant difference between PS and P; € = denotes a significant difference between P and C; ¥:*p* ≤ 0.05; ¥¥¥:*p* ≤ 0.001; £:p ≤ 0.05; ££:*p* ≤ 0.01; €:*p* ≤ 0.05

Test results are outlined in Tables 3 and 4. After the intervention, PS showed significant improvements of all sprint times relative to C and P, with no significant differences between P and C. Change-of-direction times were also shortened for PS relative to P and C (Table 3, Fig. 1). P also showed improvement relative to C on the Illinois-MT. Both plyometric groups showed similar increases in vertical jump performance (PS: SJ: ∆ 30.1%, *p* ≤ 0.001; CMJ: ∆ 39.7%, *p* ≤ 0.01; P: SJ: ∆ 30.9%, p ≤ 0.001; CMJ: ∆ 39.7%, p ≤ 0.01).

**Table 3:** Comparison of sprint, change of direction and jump performance between groups before and after the 7-week trial

Variables	Group	Pre-trial	Post-trial	*p* value	d (Cohen)
***Sprint***					
5 m (s)	PS	1.21 ± 0.11	0.99 ± 0.12 ¥¥¥ ££	< 0.001 a	1.21
	P	1.22 ± 0.06	1.14 ± 0.08	< 0.001 b	1.38
	C	1.22 ± 0.05	1.21 ± 0.04	< 0.001 c	1.12
10 m (s)	PS	2.17 ± 0.14	1.68 ± 0.23 ¥¥¥ ££	< 0.001 a	1.40
	P	2.14 ± 0.12	2.01 ± 0.11	< 0.001 b	1.55
	C	2.16 ± 0.09	2.14 ± 0.10	< 0.001 c	1.50
20 m (s)	PS	3.57 ± 0.25	3.14 ± 0.11 ¥¥ £	0.005 a	0.90
	P	3.58 ± 0.22	3.40 ± 0.12	< 0.001 b	1.17
	C	3.55 ± 0.20	3.54 ± 0.18	0.002 c	0.98
***change-of-direction***					
T-Half (s)	PS	7.00 ± 0.30	6.37 ± 0.25 ¥¥¥ £	< 0.001 a	1.24
	P	7.17 ± 0.39	6.74 ± 0.28	< 0.001 b	1.17
	C	7.13 ± 0.36	7.14 ± 0.30	0.007 c	0.88
Illinois-MT (s)	PS	13.0 ± 0.3	11.9 ± 0.4¥¥¥ £	< 0.001 a	1.35
	P	13.0 ± 0.4	12.4 ± 0.5 €	< 0.001 b	1.55
	C	13.1 ± 0.2	13.0 ± 0.2	< 0.001 c	1.19
***Jump tests***					
SJ (cm)	PS	28.6 ± 4.0	36.6 ± 3.3 ¥¥¥	< 0.001 a	1.13
	P	27.2 ± 3.8	35.6 ± 2.5 €€	< 0.001 b	1.98
	C	27.3 ± 3.0	29.5 ± 2.7	0.005 c	0.90
CMJ (cm)	PS	29.2 ± 3.5	40.3 ± 5.3 ¥¥	0.002 a	0.98
	P	30.7 ± 3.4	39.0 ± 3.1 €€	< 0.001 b	1.93
	C	30.4 ± 3.4	31.8 ± 3.1	< 0.001 c	1.14
5JT (cm)	PS	10.4 ± 0.6	11.1 ± 0.5	0.198 a	0.48
	P	9.8 ± 1.2	11.1 ± 1.5	0.012 b	0.69
	C	10.1 ± 1.1	10.2 ± 1.2	0.238 c	0.45

PS = plyometrics on sand; P = standard plyometrics; C = control group; n = number; s = seconde; SJ = squat jump; CMJ = counter-movement jump; 5JT = five-jump test; T- Half = T-Half test; Illinois-MT = Modified lllinois test; ¥ = denotes a significant difference between PS and C; £ = denotes a significant difference between PS and P; € = denotes a significant difference between P and C; a = denotes a main effect of group, b = denotes a main effect of time; c = denote a group x time interaction; ¥: p ≤ 0.05; ¥¥:p ≤ 0.01; ¥¥¥: p ≤ 0.001; £: p ≤ 0.05; ££: p ≤ 0.01; £££: p ≤ 0.001; €: *p* ≤ 0.05; €€€: *p* ≤ 0.001

**Table 4:** Comparison of repeated sprint T-test and balance performance between groups before and after the 7-week trial

Variables	Group	Pre-trial	Post-trial	*p* value	d (Cohen)
***Repeated sprint T-test***					
Repeated sprint T-test–Best time (s)	PS	12.2 ± 0.6	10.2 ± 0.6 ¥¥¥ £	< 0.001 a	1.47
	P	12.2 ± 0.3	11.2 ± 0.5 €	< 0.001 b	1.99
	C	12.2 ± 0.6	12.1 ± 0.5	< 0.001 c	1.43
Repeated sprint T-test-Mean time (s)	PS	12.5 ± 0.5	10.5 ± 1.0 ¥¥	0.003 a	0.97
	P	12.4 ± 0.3	11.0 ± 1.1 €	< 0.001 b	1.58
	C	12.4 ± 0.7	12.3 ± 0.6	≤0.001 c	1.04
Repeated sprint T-test–Fatigue index	PS	−4.4 ± 2.1	−2.4 ± 0.6 ¥¥¥	< 0.001 a	1.40
	P	−4.0 ± 2.1	−3.7 ± 2.3 €€€	0.006 b	0.76
	C	−8.5 ± 3.5	−5.6 ± 3.0	0.199 c	0.48
Repeated sprint T-test–Total time (s)	PS	87.5 ± 3.9	73.7 ± 7.1 ¥¥	0.003 a	0.97
	P	86.9 ± 2.3	77.0 ± 8.1 €	< 0.001 b	1.58
	C	87.0 ± 4.8	86.5 ± 4.3	≤0.001 c	1.04
***Y Balance Test***					
Right support leg					
RL/L (cm)	PS	83.6 ± 6.6	98.1 ± 12.7 ¥	0.023 a	0.75
	P	83.6 ± 7.8	94.0 ± 9.3	0.000 b	1.13
	C	82.2 ± 6.2	84.9 ± 6.1	0.087 c	0.60
RL/B (cm)	PS	106.3 ± 6.4	122.5 ± 7.0 ¥¥¥	< 0.001 a	1.55
	P	104.6 ± 5.0	122.5 ± 8.9 €€€	< 0.001 b	1.98
	C	102.0 ± 6.1	105.2 ± 5.5	≤0.001 c	1.03
RL/R (cm)	PS	51.4 ± 9.9	55.7 ± 9.3	0.894 a	0.12
	P	52.3 ± 10.8	55.5 ± 11.0	0.335 b	0.26
	C	52.1 ± 12.1	52.6 ± 12.1	0.843 c	0.15
**Left support leg**					
LL/L (cm)	PS	48.9 ± 8.7	53.3 ± 7.5	0.702 a	0.22
	P	51.0 ± 12.2	54.9 ± 9.6	0.112 b	0.43
	C	51.7 ± 9.4	55.1 ± 9.1	0.987 c	0.000
LL/B (cm)	PS	110.7 ± 5.8	122.1 ± 8.7 ¥¥¥	< 0.001 a	1.12
	P	103.3 ± 3.5	118.5 ± 8.1	< 0.001 b	1.29
	C	104.7 ± 9.9	107.5 ± 9.7	0.048 c	0.67
LL/R (cm)	PS	86.5 ± 6.7	90.7 ± 6.5	0.169 a	0.51
	P	84.3 ± 6.8	90.7 ± 5.5	0.013 b	0.68
	C	82.2 ± 9.5	86.3 ± 9.6	0.865 c	0.14
***Stork Balance Test***					
Right leg (s)	PS	3.25 ± 1.36	15.12 ± 2.07 ¥¥¥ ££	< 0.001 a	1.49
	P	5.11 ± 5.44	6.76 ± 5.56€	< 0.001 b	1.13
	C	3.18 ± 1.31	3.54 ± 1.62	< 0.001 c	1.61
Left leg (s)	PS	4.41 ± 3.07	15.18 ± 3.07 ¥¥¥ £££	< 0.001 a	2.26
	P	4.43 ± 3.38	5.42 ± 4.54 €€	< 0.001 b	1.44
	C	2.07 ± 0.53	2.41 ± 0.72	< 0.001 c	1.73

PS = plyometrics on sand; P = standard plyometrics; C = control group; RL = right leg; LL = left leg; L = left; R = right; B = back; n = number; s = seconde; ¥ = denotes a significant difference between PS and C; £ = denotes a significant difference between PS and P; € = denotes a significant difference between P and C; a = denotes a main effect of group, b = denotes a main effect of time; c = denote a group x time interaction; ¥: p ≤ 0.05; ¥¥:p ≤ 0.01; ¥¥¥: p ≤ 0.001; £: *p* ≤ 0.05; ££: p ≤ 0.01; £££: *p* ≤ 0.001; €: *p* ≤ 0.05; €€€: *p* ≤ 0.001

**Fig. 2:**
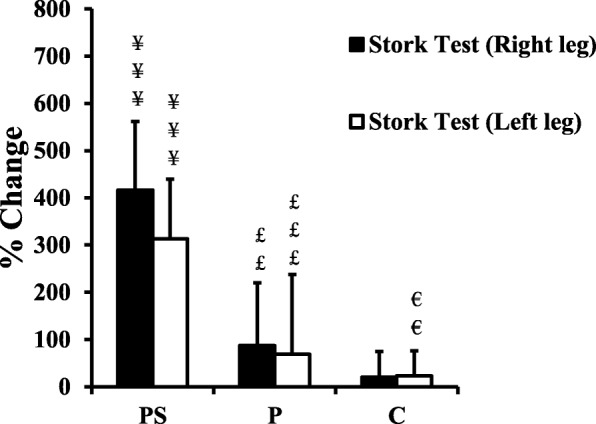
Training associated changes in dynamic balance test (Stork balance test) following plyometric training on sand (PS), plyometric training on a stable surface (P) and controls following the standard regimen (C). ¥ = denotes a significant difference between PS and C; £ = denotes a significant difference between PS and P; € = denotes a significant difference between P and C; ¥:p ≤ 0.05; ¥¥¥:p ≤ 0.001; ££:p ≤ 0.01; £££:p ≤ 0.001; €:p ≤ 0.05; €€:p ≤ 0.01

Scores on the 5-jump test remained unchanged for all groups. RSTT scores showed gains for both P and PS with respect to best time, mean time, and total time, but PS demonstrated a greater improvement in best times than P (Table 4, *p* < 0.05). Stork balance scores increased in PS relative to P and C, with P also showing a gain relative to C (left leg, p ≤ 0.01) (Fig. 2). PS yielded gains of Y-balance in 2 of 3 scores for the right leg and 1 of 3 scores for the left leg test relative to C, whereas only scores for the right leg/back (RL/B, *p* ≤ 0.001) were increased in P relative to C.

## Discussion

The major empirical finding from the present study is that experienced adolescent handball players who are exposed to a particular level of plyometric training, show enhanced gains of sprinting, change-of-direction, and static balance when this training is performed on sand rather than a gymnasium floor surface. However, other studies with differing participant groups and intensities of plyometric training have not always observed such benefits.

How could an unstable surface affect the response to plyometric training? A resulting decrease in ground reaction times [8], with increased lateral movement and balancing might increase biomechanical learning, neuromuscular adaptations [4] and strengthen the muscles involved in balancing, thus enhancing the training response seen on firm ground. Indeed, when jumping onto the sandy surface, the foot descends into the sand, causing the athlete to activate an additional force to make successive jumps and over time this seems likely to enhance strength.

Several investigators have emphasized the potentially favorable influence of training on an unstable surface upon balance and agility [7, 8, 18, 19], offering as it does specific training in the challenges faced during actual play on uneven and soggy fields. However, depending on the age, maturity and training status of athletes, the likelihood that the sandy surface corrects some overtraining may also be foreshadowed by the increase of muscle strength seen with the tapering of a standard plyometric regimen [20]. In support of this idea, Impellizzeri et al., [10] noted that when their plyometric training programs were conducted on sand, muscle soreness was reduced, and Miyama et Nisoka [21] had similar findings. If a correction of overtraining is indeed a factor, the extent of the enhancement of performance observed on switching to a sandy surface would depend on the interval between the final training session and the test measurements (7–9 days in the present study). This issue could perhaps be clarified by experimenting with various intensities of plyometric activity, and taking careful note of sensations of muscle soreness and the intramuscular leakage of marker enzymes such as creatine kinase [10, 21–24].

It could be explained that increases in sprint performance in both plyometric groups in our study reflect increases in muscle strength and power [4, 7–9, 25]. But in contrast to the present data, Dello Iacono et al. [1] found significant gains in 10-m performance with 8 weeks of plyometric training on either a firm floor or two highly unstable surfaces (∆ 1.5 and 1.9%, respectively; both *p* < 0.05); their study also showed a trend toward similar gains in 30-m performance (∆ 0.7 and 0.9%, respectively; *p* = 0.08) [7]. Likewise, Negra et al. [6] observed rather similar improvements in sprinting after pre-pubertal soccer players underwent 8 weeks of either unstable (0–10 m [∆6%], 0–20 m [∆5%], *p* < 0.01) or stable (0–10 m [∆4%], 0–20 m [∆4%], p < 0.01) plyometric training. The difference that we observed between PS and the other two groups (P and C) is explained by the fact that our athletes were in a period of physical preparation (pre-season), and had not yet reached peak form.

The present study found greater gains in the ability to change direction rapidly (an important asset for handball players [26] from training on sand (T-Half ∆ 8.9%; Illinois-MT ∆8.3%) rather than a stable surface (T-Half 5.8%∆; Illinois-MT ∆4.2%) surfaces. Arazi et al. [8] also found positive effects of depth jump training on sand vs. land surface on change-of-direction T test performance in healthy men. On the other hand Negra et al. [6] demonstrated similar improvements in the Illinois-MT score following 8 weeks of plyometric training on either a stable (Δ3%, *p* < 0.01) or an unstable surface (Δ3%, *p* < 0.01). Likewise, Granacher et al. [7] observed similar improvements in change-of-direction abilities (Δ2.9 to 3.1%, both *p* < 0.001) in sub-elite adolescent male soccer players after 8 weeks of plyometric training on either stable or unstable surfaces Any increase in change-of-direction performance of PS relative to P could be explained by the fact that athletes must develop a higher force to clear hurdles when exercising on a sand surface. During the jump on the sand, the foot sinks into the sand, and the athlete must exert an additional force to perform a succeeding jump [7, 8, 10]. Over time, the body adjusts to this greater demand, improving its strength thorough an increased nerve conduction velocity, a maximizing of the electromyogram, improved inter-muscular coordination, an enhanced motor unit recruitment strategy, and an increased excitability of the Hoffman reflex (H-reflex), as well as by changes in muscle size and architecture, and single-fibre mechanics [4, 9, 19]. During a plyometric movement, the muscles switch rapidly from an eccentric to a concentric phase of contraction [4]. A decreased duration of the amortization phase exploits stored elastic energy and the stretch reflex, allowing a greater than normal release of power during the concentric phase of movement; possibly, this phenomenon is improved more by PS than by P [7, 8, 10].

In terms of jumping ability, the current investigation showed similar increases in SJ, CMJ and 5-jump scores for PS and P. Likewise, Negra et al. [6] saw similar improvements in the standing long jump of pre-pubertal soccer players on stable (∆6%, *p* < 0.01) and unstable surfaces (∆6%, p < 0.01), and Granacher et al. [7] actually found larger gains in counter-movement jump height of young male soccer players (∆12.9%, p < 0.01) after 8 weeks of plyometric training on stable rather than unstable surfaces. However, Mirzaei et al. [27] observed increases in vertical and standing long jumps after both drop-jump and counter-movement jump when training on sand. Arazi et al. [8] also found that sand-based depth-jump training enhanced jump performance more than land-based depth-jump training.

A number of previous studies have underlined the benefit from balance training programs [6, 7, 28] and the unstable nature of the sand surface may be helpful in developing this ability. Certainly, PS enhanced Stork Stand scores relative to P in our study; this difference may reflect the fact that PS training strengthened the tendons and ligaments, thereby improving stork balance test performance. Negra et al. [6] also observed gains of Stork Balance scores in pre-pubertal soccer players (∆121 and 149%, both *p* < 0.01) after 8 weeks of plyometric training on unstable surfaces, although Granacher et al. [7] did not detect any significant difference of response in adolescent soccer players between stable and unstable plyometric training.

## Conclusions

Junior male handball is becoming progressively more athletic and is making ever greater physical demands on participants. However, players also need strength and power to win a running or jumping duel or to catch the ball before their opponents. Our results, conducted on junior handball players at a crucial point during their pre-competitive preparation, suggest that a 7-week training program of either PS or standard P improves jump, repeated change-of-direction, and static balance performance. However, PS seems to induce some additional gains of athletic performance not seen with standard P, particularly in terms of sprint, change-of-direction, repeated sprint T-test (best time), and static balance. Thus, coaches should be encouraged to include PS as an element of in pre-season conditioning.

## Data Availability

The data collected and analysed in the present study are not publicly available due to ethical restrictions but are available from the corresponding author upon request.

## Abbreviations

PS: Plyometrics on sand
P: Standard plyometrics
C: Control group
n: number
SJ: Squat jump
CMJ: Counter-movement jump
5JT: Five-jump test
T- Half: T-Half test
Illinois-MT: Modified lllinois
RL: Right leg
LL: Left leg
L: Left
R: Right
B: Back
